# Pathological axes of wound repair: Gastrulation revisited

**DOI:** 10.1186/1742-4682-7-37

**Published:** 2010-09-14

**Authors:** Maria-Angeles Aller, Jose-Ignacio Arias, Jaime Arias

**Affiliations:** 1 Surgery I Department. School of Medicine. Complutense University of Madrid. Madrid. Spain; 2 General and Digestive Surgery Unit. Monte Naranco Hospital. Consejeria de Salud y Servicios Sanitarios, Principado de Asturias. Oviedo. Spain

## Abstract

Post-traumatic inflammation is formed by molecular and cellular complex mechanisms whose final goal seems to be injured tissue regeneration.

In the skin -an exterior organ of the body- mechanical or thermal injury induces the expression of different inflammatory phenotypes that resemble similar phenotypes expressed during embryo development. Particularly, molecular and cellular mechanisms involved in gastrulation return. This is a developmental phase that delineates the three embryonic germ layers: ectoderm, endoderm and mesoderm. Consequently, in the post-natal wounded skin, primitive functions related with the embryonic mesoderm, i.e. amniotic and yolk sac-derived, are expressed. Neurogenesis and hematogenesis stand out among the primitive function mechanisms involved.

Interestingly, in these phases of the inflammatory response, whose molecular and cellular mechanisms are considered as traces of the early phases of the embryonic development, the mast cell, a cell that is supposedly inflammatory, plays a key role.

The correlation that can be established between the embryonic and the inflammatory events suggests that the results obtained from the research regarding both great fields of knowledge must be interchangeable to obtain the maximum advantage.

## Introduction

Inflammation is considered the fundamental scientific principle underlying the practice of surgery [1]. Although nowadays the main role of the inflammatory response is due to its close relationship with illness and therefore is pathological, maybe the origin of these mechanisms have a different meaning, even physiological. Thus, we have previously proposed that the evolutive phases of the post-traumatic inflammatory response may have a trophic meaning for the injured tissue [2]. Based on this supposition it would not be unreasonable to consider most of the inflammatory mechanisms as remnants of ancestral times when life depended on their trophic activity [3]. Fortunately, these mechanisms do not only represent remnants from the past in the case of injury, but also assume their ancient phenotypes in favor of survival [2,3].

When acute tissue damage is produced by a mechanical or thermal harmful stimulus, both types of energy are etiologically involved, either in tissue injury production, usually a wound [4], or in triggering an inflammatory response [5]. Cellular lesions are irreversible in the wounds produced by mechanical and thermal energy since necrosis is produced [5]. Until recently, necrosis has often been viewed as an accidental and uncontrolled cell death process. Nevertheless, growing evidence supports the idea that necrotic cell death may also be programmed [6]. Cellular signaling events have been identified to initiate necrotic destruction that could be blocked by inhibiting discrete cellular processes [7].

The most relevant mechanisms culminating in cell necrosis correspond to mitochondrial dysfunction and ATP depletion; loss of intracellular ion homeostasis, with osmotic swelling and oxidative stress; activation of degradative hydrolases, and degradation of cytoskeletal proteins with disruption of cytoskeletal integrity [8]. Surprisingly enough, this list of mechanisms also correspond to what occurs in the acute inflammatory post-injury response [2,3]. It seems that, in response to injury, cells can develop mechanisms that would play a defensive role, i.e. inflammation, and which could favor reversing the alterations until their inadequate expression would make them harmful, i.e. cell death [9]. Hence, at a specific moment in time, the pathophysiological mechanisms, i.e. cellular response to injury, become a pathogenic mechanism, i.e. producers of cell death [3]. Thus, it could be considered that the cells can "escape" death in attacked tissues. Taken all together these mechanisms would in turn constitute the post-injury inflammatory response [2,3,10].

### Wounds and Inflammation

**Figure 1:**
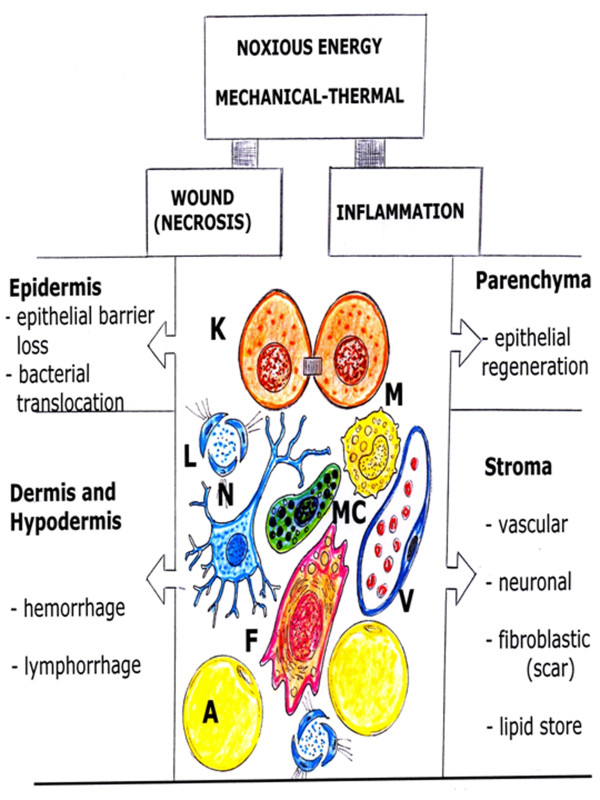
**Consequences of noxious -mechanical and thermal energy- over the skin organ, that is formed by epidermis (parenchyma), and dermis and hypodermis (stroma)**. A: Adipocyte; F: Fibroblast; K: Keratinocyte; L: Lymphatic capillary; M: Macrophage; MC: Mast cell; N: Neuron; V: Post-capillary venule;

The skin is protecting the organism against physical, chemical and microbial impacts of the environment [11,12]. It represents the second largest organ in adult humans, only surpassed by the vascular system [12]. The skin, consists of an outer squamous epithelium, the epidermis and its appendages (sweat glands, pilosebaceous follicles and nails) and two inner layers of connective tissues, the dermis and the hypodermis [11,13]. Therefore, a wound that includes the three layers of this organ would injure its parenchyma, or epidermis, and the stroma, which is made up of dermis and hypodermis (Figure 1).

The inflammatory response expressed by this organ after a wound can have exogenous and endogenous inducers [9]. Noxious mechanical or thermal stimuli as exogenous signals and cellular necrosis, as endogenous signals, can initiate the inflammatory response [14,15]. Thus, mechanical or thermal energy, as an exogenous damage/alarm signal [14,15], have the ability to produce a wound, i.e. damage, as well as initiate an inflammatory response, i.e. alarm.

Today, the role that inflammation *"per se" *plays in cutaneous wound repair is most likely very limited. Thus, it is accepted that inflammation is only another component of the repair process. Thus, the common description of wound repair evolution includes three classic types: Inflammation, new tissue formation and remodeling [16-19]. However, some authors describe four healing phases: Hemostasis, inflammation, repair and remodeling [20] and even five phases: Hemostasis, inflammation, cellular migration and proliferation, protein synthesis and wound contraction and remodeling [21].

Nowadays, we need integrative pathophysiology to integrate all the new knowledge to understand the inflammatory response because the distance between new molecular knowledge and every day patient care is increasing. Now we need to understand cell biology and genetics of inflammation better to identify gene and metabolic targets in order to modulate aspects of the inflammatory response [22]. We have therefore proposed that the inflammatory wound response recapitulates ontogeny and phylogeny through trophic mechanisms of increasing complexity to the injured tissue [2,10].

### Phases and phenotypes during wound repair

The inflammatory response that is induced in the injured tissue could be described as a succession of three overlapped phases, during which the phenotypes of metabolic progressive complexity related to the use of oxygen are expressed. Each one of these phases emphasizes the trophic role of the mechanisms developed in the damaged tissue. Hence, nutrition by diffusion predominates the first phase; trophism is mediated by inflammatory cells in the second phase; and finally blood circulation and oxidative metabolism play the most significant nutritive roles in the third phase [10].

**Figure 2:**
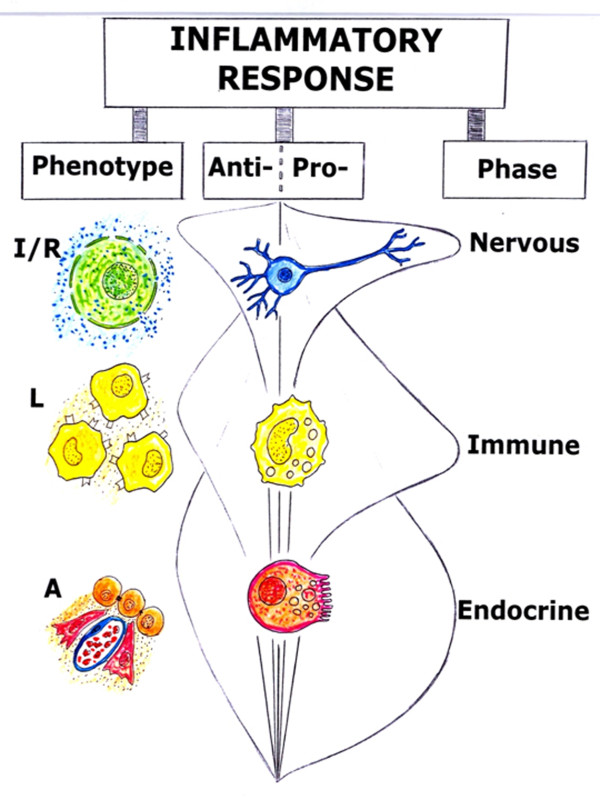
**The inflammatory response which is developed after skin injury is divided into evolutive vascular phenotypes and phases**. Ischemia-reperfusion (I/R), leukocytic (L) and angiogenic (A) phenotypes are successively expressed during the vascular inflammation. The injured tissue losses its normal structure and acquires functional autonomy during ischemia-reperfusion and leukocytic phenotype expression. Then, when the angiogenic phenotype is progressively expressed, the tissue is re-structured and specialized. In the immediate nervous phase, depolarization and repolarization of cell membranes would be the key pathophysiological mechanism. During the immune phase, the transient synthesis of adhesion molecules favors cellular and bacterial translocation. Lastly, in the endocrine phase the skin tries to recover its parenchymatous structure, or epithelium (regeneration), as well as its stroma or connective tissue (scarring).

Since these trophic mechanisms are of increasing complexity, progressing from anoxia to total specialization in the use of oxygen to obtain usable energy, it could be speculated that they represent the successive reappearance of the stages that took place during the evolution of life without oxygen on Earth from ancient times. In this sense, the inflammatory response not only could recapitulate phylogeny, but also ontogeny, through the successive expression of phenotypes that have a trophic meaning for the injured tissue [2,3,10] (Figure 2).

The successive inflammatory phenotypes are expressed mainly in the interstitial space. Therefore, the interstitial space always seems to be the battlefield for inflammation, whether it is due to trauma [2,3], infection [3] or tumors [23-25].

In the first or immediate phase of the inflammatory response, interstitial hydroelectrolytic alterations stand out. This phase has been referred to as the nervous phase, because the sensory (pain and analgesia) and motor alterations (contraction and relaxation of smooth and skeletal muscle fibers) respond to the injury. Particularly, the vasomotor response -with vasoconstriction and vasodilation- is responsible of the ischemia-reperfusion phenomenon, with the subsequent excessive production of reactive oxygen and nitrogen species (ROS/RNS) that causes oxidative and nitrosative stress in the injured tissue. In this phase, during the progression of the interstitial edema, the space between epithelial cells and capillaries increases, and the lymphatic circulation is simultaneously activated (circulatory switch) [2,10].

In the following intermediate or immune phase of the inflammatory response, the tissues which have undergone ischemia-reperfusion suffer an immunological activation. In addition, they are infiltrated by inflammatory blood-born cells, particularly leukocytes. In order to infiltrate the interstitial space, bacteria takes advantage of the chemotactic call, which activates and induces the recruitment of blood cells. In the tissue which suffers oxidative and nitrosative stress, symbiosis of the leukocytes and bacteria for extracellular digestion by enzyme release, i.e. fermentation, and by intracellular digestion, i.e. phagocytosis, produces enzymatic stress. Furthermore, macrophages and dendritic cells take advantage of the lymphatic circulation activation, and migrate through it until reaching the lymph nodes, where they activate lymphocytes [2,3].

During the third phase of the inflammatory response, angiogenesis permits numerous substances, including hormones, to be transported by the blood circulation. For this reason, it has been considered that the predominance of angiogenesis during the last phase of the inflammatory response would allow for calling it the endocrine phase. Although the final objective of the angiogenic phenotype is to form new mature vessels for oxygen, substrates and blood cells, other functions could be carried out before the new mature vessels are formed. Thus, angiogenesis could have antioxidant and antienzymatic properties, favoring the resolution of the inflammation as well as wound repair by epithelial regeneration and scarring. Therefore, in this phase the new formed tissue is structured, specialized and matures by remodeling [2,3,10] (Figure 2).

The three overlapped trophic phases of the post-traumatic inflammatory response could also be named, by their corresponding length, as acute, subacute and chronic, respectively. The acute phase is characterized by the quick molecular infiltration of the interstitial space that would for favoring the establishment of a trophic axis based in the interstitial fluid flow. In the following or subacute phase, the cellular infiltration of the interstitial space predominates. In this phase, the invasion of the interstitium by blood cells would create another trophic axis based on a hypothetical enzymatic digestive ability that is assumed by the leukocytes in the injured tissue. Finally, it could be interpreted that through the confluence in the interstitial space of both trophic axes, molecular and cellular, the appropriate metabolic conditions would be generated so that tissue repair takes place during the last so-called chronic phase of the inflammatory response.

### Embryonic bases of inflammation: The amnion and the yolk sac

The inflammatory response could recapitulate ontogeny through the expression of the two hypothetical trophic axes, molecular and cellular, in the interstitial space of the injured tissue.

We have previously proposed the hypothesis that inflammation would represent the debut during post-natal life of ancestral biochemical mechanisms that were used for normal embryonic development. The re-expression of these old mechanisms, with a prenatal solvent path, are perhaps inappropriate and hard to recognize since they are anachronistic during post-natal life and because they are established in a different environmental medium [3,26].

**Figure 3:**
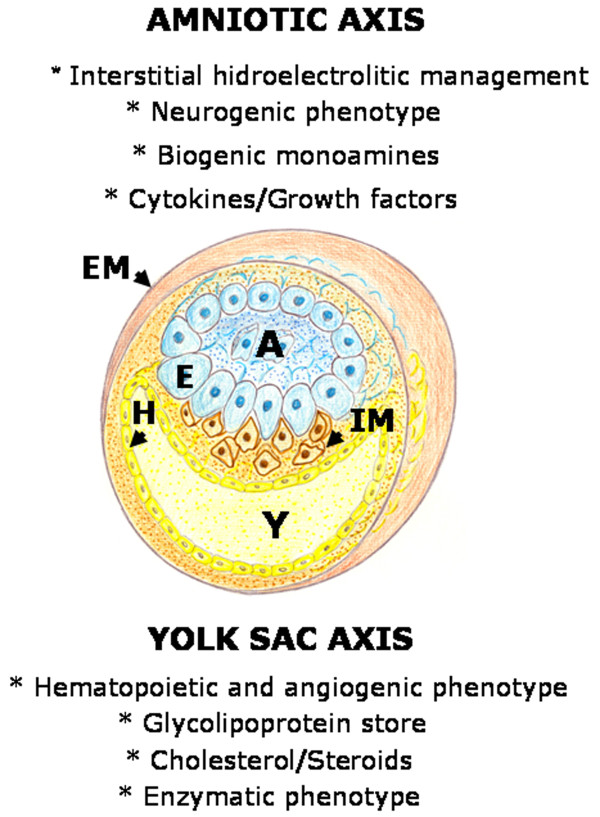
**Schematic representation of the early mammalian embryo during gastrulation**. The extraembryonic mesoderm (EM) is represented surrounding the amniotic cavity (A) and the yolk sac cavity (Y). Between the epiblast (E) and the hypoblast (H) the mesoderm internalizes (IM) by epithelial-mesenchymal transition. On top of the figure, some of the characteristics of the amniotic axis are summarized. On the other hand, on the bottom, some of the characteristics of the yolk sac axis are exposed.

The early mammalian embryo already has the ability to manage fluids in the interstitial space. In the human blastocyst, the inner cell mass or embryoblast, differentiates into two layers, the hypoblast and epiblast. The epiblast is the source of all three germ layers and develops within a small cavity named amniotic cavity [27]. At the early stages of pregnancy, amniotic fluid consists of a filtrate of maternal blood. That is why in this period drugs taken by the mother can enter the amniotic fluid by diffusion across the placenta [28]. Amniotic fluid is an essential component for fetal development and maturation during pregnancy [29]. During these stages, amniotic fluid is a bioactive medium actively secreted by the cells lining the amniotic cavity and as gestation progresses it includes significant volume of fetal urine [30] (Figure 3).

Body fluid is distributed among three major fluid spaces: Intracellular fluid, interstitial fluid and plasma. Nevertheless, the fluid distribution in each of these compartments is dramatically different in the fetus compared to the adult. Particularly, the amniotic fluid that surrounds the fetus may be considered an extension of the extracellular space of the fetus [31]. Thus, the lymphatic system plays an essential role in the regulation of fluid distribution between the plasma and the interstitial fluid and, probably with the amniotic fluid [31]. It could also be hypothesized that similar functions, i.e. development and maturation, that the amniotic-lympathic-interstitial fluid axis has in the embryo, could have interstitial edema and activated lymphatic circulation (circulatory switch) in the traumatized tissue.

The yolk sac is the final destination of migrating visceral endoderm cells, that in turn are derivatives of the hypoblast (Figure 3). The yolk sac begins to form during gastrulation [32]. The visceral yolk sac expands and blood islands -structures consisting of hematopoietic progenitors surrounded by a loose network of endothelial cells- appear [32]. Endothelial cell precursors associated with blood islands differentiate and coalesce to form a primitive circulation bed, which later connects to the embryo via the vitelline vessels [32,33].

**Figure 4:**
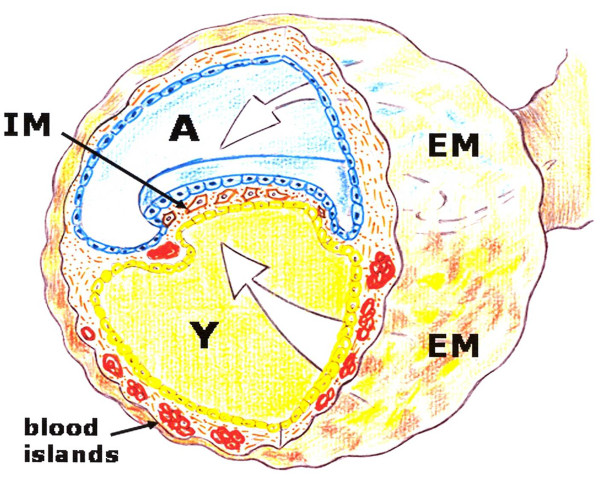
**Schematic representation of the gastrulating embryo under the hypothetical influence of an amniotic-interstitial fluid-neurogenic axis (A) and yolk sac-hematopoietic-angiogenic axis (Y)**. EM. Extraembryonic mesoderm. IM: intra-embryonic mesoderm.

Mammalian development requires the rapid *de novo *formation of embryonic blood cells to support embryonic and fetal growth prior to the establishment of the adult hematopoietic system. The very first blood cells to appear in the embryonic circulation arise in the extraembryonic yolk sac [34]. Particularly, primitive macrophages first develop in the yolk sac [35]. As the embryo develops, newly formed hematopoietic stem cells are found in the aorta-gonad-mesonephros region, then in the fetal liver, thymus and spleen and lastly, for adult hematopoiesis, in the bone marrow [36]. The yolk sac suffers a rapid involution following completion of their hemopoietic and angiogenic functions [37] (Figure 4).

Thus, the yolk sac-hematopoietic-angiogenic axis coordinates blood cell differentiation and vessel formation and plays an essential role supporting embryonic and fetal growth [32]. It could also be hypothesized that a similar trophic cellular axis is developed by the traumatized patient.

**Figure 5:**
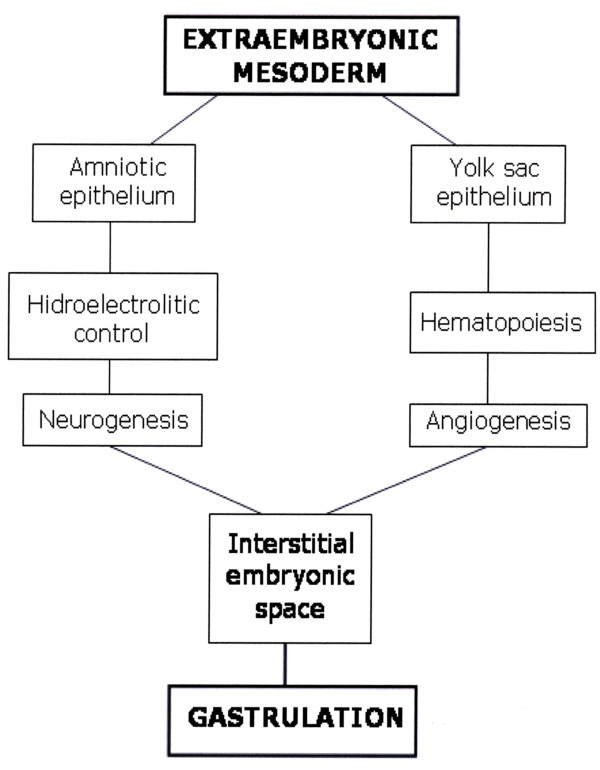
**Hypothetical embryonary interstitial confluence of amniotic and yolk sac trophic axis to induce gastrulation**.

The confluence during mammalian development of the amniotic-interstitial fluid axis and the yolk sac-hematopoietic-angiogenic axis could be one essential factor to drive gastrulation (Figure 5). Although the details of gastrulation differ among different species, the cellular mechanisms involved in gastrulation are common to all animals. After induction of the germ layers, the blastula, composed by pluripotent stem cells, is transformed by gastrulation movements into a multilayered embryo, including ectoderm, endoderm and mesoderm with head, trunk and tail rudiments [38]. During the internalization process, cells of the mesoderm move through the blastopore under the ectoderm. Mesoderm, the middle or interstitial germ layer, gives rise to hematopoietic, endothelial, heart, skeletal muscle and connective tissues [38].

In the current review we suggest that in the traumatized tissue both rudimentary and hypothetical trophic axes could be re-expressed with a similar aim: the creation of a specialized tissue once again. If so, probably during damaged tissue repair, a similar set of morphogenetic cell behaviors are used as in gastrulation, including a series of changes in cell motility, cell shape and cell adhesion. This post-traumatic recreation of embryonic processes in the adult tissues is a hypothesis that would be supported by the recent findings about pluripotent stem resident cells in specialized tissues [36,39]. Stem cells with pluripotent/multipotent capacity were thought to be restricted to the early embryonic stages. However, recent evidence challenged this idea by confirming the presence of pluripotent/multipotent stem cells in adult tissues and organs. These cells may participate in cellular turnover and the rebuilding pool of the tissue circumstances such as tissue injury [39]. However, their expression in the post-natal body under the influence of multiple anomalous environmental factors could induce pathological actions associated with tissue reparation.

**Figure 6:**
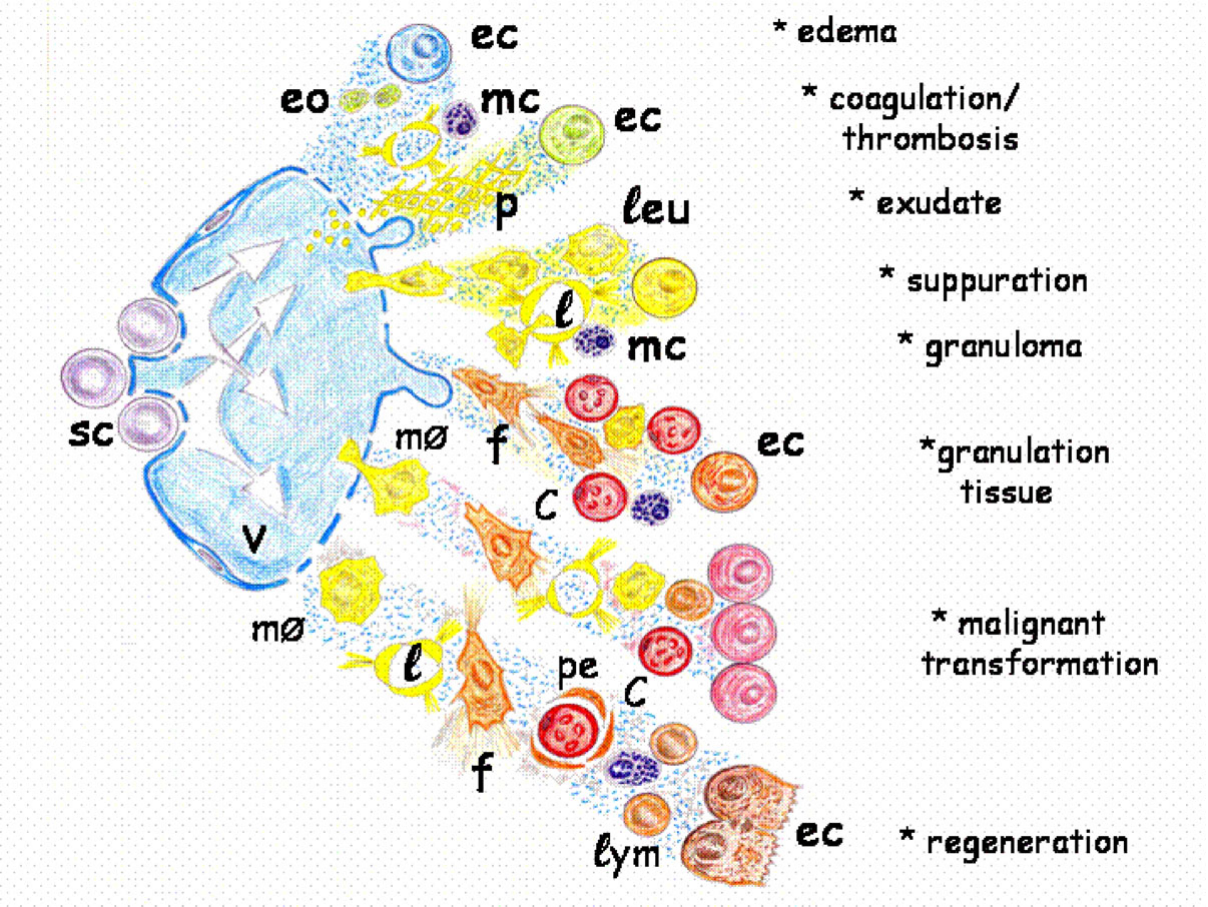
**Schematic representation of the pathological axes expression during vascular inflammation, which is developed in the interstitial space of the injured tissue**. c: capillary; ec: epithelial cell; eo: eosinophils; f: fibroblast; l: lymphatic vessel; leu: leucocytes; lym:lymphocyte; mc: mast cell; mØ: macrophage; p: platelets and fibrin; pe: pericyte; sc: stem cell; v: post-capillary venule.

It has been accepted that the impairments or pathologies associated with wounded tissue repair during post-natal life have an interstitial origin because it is supposedly in this space, which is successively occupied by the mesoderm and then, by the connective tissue, where the hypothetical embryonic trophic axes are re-expressed after trauma. Maybe, this is the reason why the alterations are common in terms of those structures that occupy the tissue space equivalent to the interstitial space, known as the stroma. Particularly, the vascular, blood and lymphatic, and nervous inflammatory alterations stand out. These inflammatory changes have been grouped for their study in pathological axes that are predominantly expressed in the interstitial space (Figure 6).

Given the confluence of the two rudimentary trophic axes in the injured tissue, we have supposed that the underlying intention is to carry out a similar phenomenon to gastrulation. Therefore, it is possible that the resulting vasculo-nervous inflammatory alterations represent morphogenetic processes related to this crucial step in early embryogenesis.

### Pathological axes of inflammation

The inappropriate expression of the inflammatory phenotypes induces evolutive post-traumatic complications. Normally, the abnormal post-traumatic inflammatory phenotypes are predominantly expressed in the vasculo-nervous structures and, for this reason, their pathophysiological mechanisms play the main role in the study of wound repair complications.

#### Pathological axis of vascular inflammation

The concept of the inflammatory response as distributed in the successive phases of ischemia-reperfusion, leukocytic infiltration and angiogenesis [40], is based on the normal microcirculatory, lymphatic and blood changes suffered by the injured tissue. Consequently, the inappropriate expression of these vascular inflammatory phenotypes, either by excess or by defect, is an obligatory reference (Figure 6).

##### * Edema

The abnormal expression of the ischemia-reperfusion phenotype during repair can produce disturbances in ion transport associated with cellular dysfunction.

There is increasing evidence that conditions characterized by an intense local inflammatory response are associated with abnormal ion transport [41]. Inflammatory mediators which influence ion transport are bradykinin, leukotriens, cytokines, thrombin, and transforming growth factor (TGF). They trigger the release of specific messengers, like prostaglandins, nitric oxide and histamine which alter the ion transport function through specific receptors, intracellular second messengers and protein kinases [41]. It has been stated that small fluctuations in cell hydration or cell volume act as a potent signal for cellular metabolism and gene expression [42]. Also, the exposure of cells to higher osmolarity results in the secretion of pro-inflammatory cytokines and extends normal macrophage half-life [43].

Interstitial edema causes a steady separation of the cells from the capillaries and widens the diffusion distance for oxygen and nutrients, favors the insufficiency of the lymphatic circulation and reduces tissue defense mechanisms leading to susceptibility to infections [3]. Also, interstitial flow is important for lymphangiogenesis [44]. The interstitial fluid flow associated with edema, even though it can be extremely slow, can have important effects on tissue morphogenesis and function, cell migration and differentiation and matrix remodeling, among other processes [45,46]. Insights into the mechanisms linking mechanical forces to cell and tissue differentiation pathways are important for understanding many diseases, including inflammation [46]. Abnormally increased interstitial flow rates can occur during inflammation and can also trigger fibroblasts to differentiate or remodel the extracellular matrix, contributing to the development of tissue fibrosis [44,45].

The impaired function or formation of lymphatic vessels after trauma, could be associated with lymphedema, which is characterized by interstitial fluid accumulation. Lymphedema can lead to increased susceptibility to infections, impaired wound healing and chronic swelling [47].

Limiting swelling is extremely important because the injured area cannot return to normal until swelling is gone. In musculoeskeletal injuries this is best accomplished with the "RICE" technique, which involves Rest, Ice, Compression and Elevation [48].

##### * Coagulation

When post-capillary venular membranes become permeable to complex molecules, including coagulation factors, the extravasation of fluids lead to interstitial coagulation. Cellular interstitial infiltration of the injured tissue is favored by the action of intrinsic and extrinsic components of the coagulation cascades. This results in the production of thrombin, which catalyzes the conversion of fibrinogen of intravascular origin to fibrin [21,49]. In most pathophysiological situations, it seems that the activation of both coagulation and complement cascades occurs simultaneously [50]. Complement and coagulation systems are organized into proteolytic cascades which are composed of serine proteases belonging to the chymotrypsin family. An explanation for the structural and functional similarities between the clotting and complement system is that they originate from a common ancestral developmental-immune cascade [51]. Thus, the functional linkages between development, hemostasis and immunity in vertebrates would be explained [50,51].

Mechanical and thermal injuries are conditions predisposed to thrombosis [21]. The mechanisms underlying this increased tendency for thrombus formation are, in part, related to the procoagulant properties of the inflammatory mediators produced and released as a response to injury [50]. The complement system contributes significantly to thrombosis by directly enhancing blood clotting properties, by augmenting the inflammatory response, which in turn potentiates inflammation [52].

Inadequate fibrin formation is associated with impaired wound healing. Any process that removes fibrin from the wound will disrupt the formation of the extracellular matrix and consequently will also delay wound healing [53]. Mast cells strategically located in the vicinity of blood and lymph vessels as well as nerve fibers, are among the first responders to the stimuli that initiate inflammation [50,54]. While initially mast cells were identified as only participating in allergic responses, it is now clear that they participate in other phenomena such as wound healing and fibrosis [54,55]. Particularly, they contribute to hemostasis through the expression of tissue plasminogen activator and heparin production, thereby preventing uncontrolled local activation of the coagulation system [50].

##### * Exudation

Chronic wound fluid is biochemically different from acute wound fluid. Chronic wounds may produce large amounts of exudate as a by-product of inflammation that contains elevated levels of enzymes. Exudate from chronic wounds has been shown to impede or block the proliferation of key cells in the wound healing process, such as keratinocytes, fibroblasts and endothelial cells [56].

##### * Suppuration

The formation of yellow, milky-yellow, greenish yellow or white-yellow pus characterizes suppuration or purulent wound inflammation [5]. In addition to the enzymes released by granulocytes during the process of phagocytosis and bacterial killing, the bacteria themselves produce a number of exoenzymes that cause tissue destruction as well as infection localization. Particularly, almost all *Staphylococcus aureus *strains have the ability to secrete an array of enzymes including nucleases, proteases, lipases, hyaluronidase and collagenase [57]. Matrix metalloproteases would also collaborate in the development of enzymatic stress in the acute inflammatory tissue injury [58]. Pus mainly contains necrotic tissue debris and dead neutrophils. When the collection of pus is localized, an abscess is established [57].

Phagocytosis of bacteria typically accelerates neutrophil apoptosis, which ultimately promotes the resolution of infection [59]. Although neutrophil apoptosis is critical for granulocyte homeostasis and the resolution of inflammation, many pro-inflammatory molecules extend the survival of polymorphonuclear leukocytes during the initial stages of the inflammatory response. However, some bacterial pathogens alter neutrophil apoptosis to survive and thereby cause disease [60].

Colonization is defined as the presence of replicating bacteria and adherent microorganism without tissue damage. However critical colonization is a novel concept that states that the bacterial burden in the chronic wound does not elicit the typical symptoms of an infection, but delays healing [61,62].

Acute lymphangitis is often the consequence of a purulent wound inflammation. It is mostly caused by *Staphylococcus pyogenes *or *Streptococci*. Acute lymphangitis is recognizable as linear erythematous streak extending from the primary lesion, i.e. wound, toward the regional lymph nodes [63,64].

Lymph nodes are essential for the initiation of immune responses by creating an environment in which lymphocytes and antigen-presenting cells can optimally interact [65]. Antigen-presenting cells as dendritic cells, macrophages and mast cells, first reach the subcapsular sinus and then, move into the paracortex where they aggregate around high-endothelial venules [65,66]. These venules allow the active immigration of T and B lymphocytes from the blood into the paracortex compartment of the lymph node [65,67]. However, wound infection by pyogenic bacteria frequently causes nodular lymphangitis with nodular subcutaneous swelling along the involved lymph nodes [63].

##### * Granuloma

Within hours of wounding, neutrophils are attracted to the wound site, followed by monocytes, which mature into macrophages as they invade tissues [68]. In some invertebrates, mesenchymal cells, endothelial cells or fibroblast-like cells can transform into macrophages [35]. In vertebrates, macrophages are involved in modulating the inflammatory process during the pathogenesis and resolution of tissue injury and inflammation [59,69,70].

Macrophages have remarkable plasticity that allows them to efficiently respond to environmental signals and change their phenotype [35,70,71]. Three macrophage populations based on three different homeostatic activities have been proposed: Host defense, wound healing and immune regulation [71]. Foreign bodies are indigestible particles and the macrophages form a granuloma around it. In some cases macrophages can fuse with each other to form "giant cells" that encapsulate the foreign body [9].

##### * Granulation tissue and remodeling

Angiogenesis is closely associated with granulation tissue formation and remodeling as the newly forming cellular complex must be supplied with oxygen and nutrients [72]. Excessive angiogenesis participates in the formation of granulation tissue, starting about three or four days after injury. The main cell types driving the excessive generation of the new tissue are macrophages, endothelial cells, fibroblasts and keratinocytes [17]. Mast cells also play a role in coordinating neovascularization in the wound [54].

Angiogenesis is regulated by numerous "classic" factors: Vascular endothelial growth factor (VEGF), fibroblast growth factor-2 (FGF-2), transforming growth factors (TGFs), angiopoietins, platelet-derived growth factor (PDGF), and thrombospondin-1 and angiostatin. "Non classic" endogenous stimulators of angiogenesis include erythropoietin, angiotensin II, endothelins, adrenomedullin, adipokines (leptin, adiponectin), neuropeptide-Y, vasoactive intestinal peptide (VIP) and substance P [73,74]. They act in synergy to stimulate endothelial cell function during angiogenesis in tissue repair [74].

As granulation tissue forms in the healing wound, the vascular cells, intermingle with the provisional matrix, which is composed mainly of fibrin, fibronectin and vibronectin [74]. Then, the new blood vessels associated with fibroblast and macrophages replace the fibrin matrix with granulation tissue, which forms a new substrate for keratinocyte migration [17,21].

Remodeling begins two to three weeks after injury and lasts for a year or more. A framework of collagen and elastin fibers replaces the granulation tissue and produces progressive tissue sclerosis [75,76]. Finally, most of the endothelial cells, with macrophages and fibroblasts undergo apoptosis, leaving a mass that contains few cells and consists mostly of collagen and other extracellular-matrix proteins [21].

#### Pathological axis of neuronal inflammation

The inflammatory mechanisms also develop a fundamental role in the production of post-traumatic pain.

##### * Inflammatory pain

Inflammatory pain is associated with sensitization of the nociceptive system. Central sensitization represents an enhancement in the function of neurons and circuits in nociceptive pathways caused by increases in membrane excitability and synaptic efficacy. It also reduces inhibition and is a manifestation of the remarkable plasticity of the somatosensory nervous system in response to inflammation [77]. To induce central sensitization, the noxious stimulus must be intense, repeated and sustained. Peripheral tissue injury is not necessary, although the degree of noxious stimulation that produces frank tissue injury almost always induces central sensitization [77,78].

Peripheral inflammation induces a phenotypic switch in primary sensory neurons that comprises a change in their neurochemical character and properties due to alterations in transcription and translation [77]. However, it could be considered that the inflammatory pain pathogeny is phase-specific. Thus, after the initial electrical phase, with upregulation of ionic channel expression in the nociceptive circuits, which causes spontaneous neural firing [78,79], the following would be an immune phase. The leading role of this phase would be played by the cytokines and immune cells acting as pain mediators and modulators [80,81]. Lastly, in an endocrine phase, neurotrophic factors, including nerve growth factor (NGF), brain-derived neurotrophic factor (BDNF) and neurotrophins 3 and 4, would be associated with structural neural remodeling [77,82].

An immediate component of the stress response to pain is the efferent nervous response, which is mediated by the somatic motor and autonomic nervous system [83]. The somatic motor response usually consists in the withdrawal of the affected part of the body from the source of irritation. Withdrawal reflexes are the simplest centrally organized response to painful stimuli [77,84]. Furthermore, the fight-or-flight response is the behavioral response to a threat, in which the somatic motor response stands out [83].

With respect to the autonomic nervous system, both the sympathetic and parasympathetic nervous systems, participate in the acute inflammatory response [83]. Catecholamines are found in adrenergic neurons, but their highest concentration is found in the peripheral presynaptic nerve terminals. After depolarizing the stimulation of these nerves, rapid secretory release of stored catecholamines occurs. Besides the adrenomedullary chromaffin cell and neuron derived catecholamines, lymphocytes and phagocytes represent a third category of catecholamine-producing cells [85].

In addition, the inflammatory reflex of Tracery is a pathway in which the autonomic system detects the presence of inflammatory stimuli and modulates inflammation. Particularly, afferent signals are transmitted to the brain, via the vagus nerve, which activates a reflex response that culminates in efferent vagus nerve signaling termed the "cholinergic anti-inflammatory pathway" [86].

Also, by anterograde inflammatory signals, neural cells contribute to local tissue responses. Noxious stimuli cause nerves to release neuropeptides in the injured tissue, i.e. substance P and Calcitonin gen related peptide (CGRP). When released into injured tissues, neuropeptides contribute to the pro-inflammatory responses against injury. The evidence that neuropeptides contribute to inflammation includes changes in endothelial cell shape, leading to capillary leak, smooth muscle cell relaxation leading to vasodilatation, mast cell degranulation leading to histamine, serotonine, proteolytic enzymes and growth factor release, induction of inflammatory cell chemotaxis, increase expression of adhesion molecule and increase cytokine and growth factor production [87-90]. Experimental and clinical research has shown that excessive neuropeptide activity induces exuberant inflammation in hypertrophic scars [89]. Moreover, the functional impotence of the somatic motor system, which controls voluntary movements, i.e. *functio laesa*, favors vascular blood and lymphatic stasis and interstitial edema [48].

##### * Complex Regional Pain Syndrome type I

Complex Regional Pain Syndrome type I is formerly identified as "reflex sympathetic dystrophy". This syndrome is the new term for "causalgia" that always coexists with documented nerve injury [91]. Complex Regional Pain Syndrome type I causes chronic pain, skin hypersensitivity, vasomotor instability with hyperemia, swelling and trophic alterations. It develops in the extremities mostly after minimal injury and it has been proposed that most of their features are explicable by small-fiber dysfunction [92]. Ectopically generated ongoing activity in afferent fibers conducted antidromically could generate neurogenic inflammation in the peripheral tissues, consisting of arteriolar vasodilation by release of CGRP and substance P, and postcapillary venular plasma extravasation by release of substance P [93,94]. Nonetheless, these peripheral inflammatory changes cannot be seen independently of the changes in the central nervous system, including the spinal cord. Both, the central and peripheral nervous system interact with each other via afferent and efferent signals, some of which may not be electrical but hormonal signals or by changes of axoplasmic transport [94].

### The inflammatory mesenchyma: A round-trip ticket

**Figure 7:**
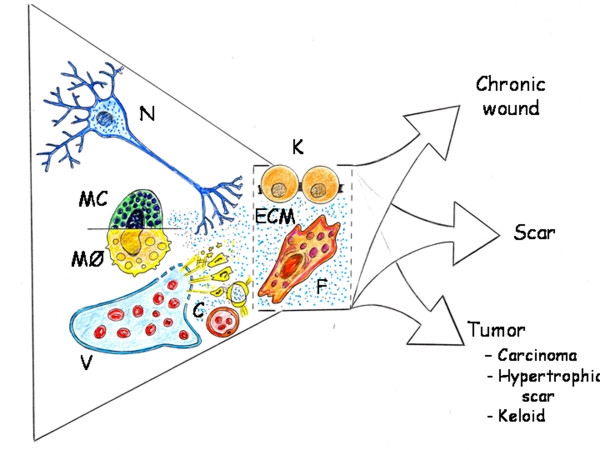
**Influence of the pathological axis of neuronal and vascular inflammation in the skin parenchyma (keratinocytes) and stroma (fibroblast)**. C: Arterial capillary; ECM: Extracellular matrix; F: Fibroblast; K: Keratinocyte; MC: Mast cell; MØ: Macrophage; N: Neuron; V: Vein;

Fetal wounds heal by regenerating normal epidermis and dermis with restoration of the extracellular matrix architecture, strength and function [22,95]. Also adult partial-thickness wounds are repaired by regeneration, i.e. replication of similar cells. In contrast, however, adult full-thickness wounds repair by connective scar tissue [56]. Therefore, the prognosis of extensive and deep wounds is not entirely satisfactory because of scar formation and the loss of normal function and skin appendages [96]. As a result, reducing the formation of scars and reestablishing the normal anatomy and function of the skin and its appendages have become the aim of regenerative medicine research [95,96] (Figure 7).

The spectrum of scarring includes normal scars, hypertrophic scars and keloids [97]. Hypertrophic scars are often red, inflamed, itchy and even painful. They generally regress spontaneously within one month to years after the initial injury [97-99]. Keloid scars are also raised, erythematous and often pruritic, however, they extend beyond the original wound boundaries and rarely regress over time [97,98,100].

Recently, the role of mast cells in wound repair and the remodeling process have been attracting attention. Mast cell proteases may provoke matrix degradation; histamine stimulates fibroblast migration and thus may contribute to wound healing regulation [101]. Particularly, keloids and hypertrophic scars have a fourfold increase in the number of mast cells compared with normal skin. In addition, hypertrophic scar mast cells release more histamine than normal skin mast cells after stimulation by substance P [102].

Although after the traumatism, vascular and neuronal inflammation are the main clinical manifestations, it must not be forgotten that the principal cells that constitute the skin organ, or parenchyma, are the keratinocytes, and the fibroblasts, whose primary function is to establish, maintain and modify the connective skin stroma [103] (Figure 7).

In the human body the fibroblasts form a heterogeneous collection of mesenchymal cells [103-105] and they are the principal cellular constituents of connective tissues [103]. Skin fibroblasts constitute a heterogeneous population of contractile (myofibroblasts, pericytes, smooth muscle cells), pro-inflammatory, highly proliferative, proangiogenic and profibrogenic cells [103,106]. Also, in the hypodermis the connective tissue has the ability to accumulate lipids, i.e. adipose cells [103].

Fibroblasts are mesodermal-derived cells and perhaps this embryonic origin could justify their great postnatal plasticity. The mesodermal cells of the embryo participate in the extraembryonary structures, including the corion, the amnios and the yolk sac [36]. Also, in the embryonic mesoderm "blood islands", develop. They consist of erythroid cells and surrounding endothelial cells which are formed in the vascular plexus of the yolk sac [36,107]. The differentiation of amnion from the epiblast occurs before gastrulation and the specification of the three germ layers and cells from the amniotic fluid show stem cell characteristics although most of the properties reported suggest that these cells are more similar to mesenchymal stem cells than to amnion epithelial cells [27]. The major role of the mesodermal cells and their ability to differentiate from the first stages of embryonic development allow for considering them as the cell protype that should be resorted to when the repair of any tissue in the body is needed [36]. And for this reason, perhaps the post-traumatic inflammatory response has the same intentions, namely, to use the embryonic mesodermal phenotype with a therapeutic objective.

**Figure 8:**
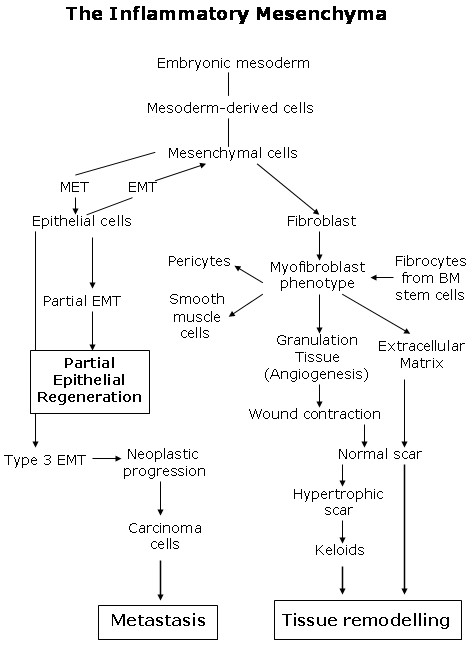
**Main role of the mesenchymal cells in the inflammatory interstitium**.

If the post-traumatic tissue repair is based on the re-expression of metabolic, histologic and functional conditions that made the first stages of embryo development possible, then the fibroblast would play a fundamental role in this repair process. In fact, this cell is recognized as a mesoderm or embryonic trace and perhaps the most qualified cell for re-expressing this original phenotype [108] (Figure 8).

Local fibroblasts residing in the skin are considered the most prominent source of myofibroblasts [106,109]. However, a variety of other precursor cells contribute to the myofibroblast population, depending on the nature of the injured tissue and the particular microenvironment [106]. Activated myofibroblasts are generated from a variety of sources, including resident mesenchymal cells, epithelial and endothelial cells via the epithelial/endothelial mesenchymal transition as well as from circulating fibroblast-like cells called fibrocytes, derived from bone-marrow stem cells [103-112]. In addition circulating monocytes have the capacity to differentiate into non-phagocyte i.e. mesenchymal cells and endothelial cells [113].

The reason why the myofibroblast is attractive to a broad scientific and clinical audience is due to the large panel of cells that can develop this phenotype upon activation. It appears that myofibroblasts can be recruited from whatever local cell type and it is suitable for rapidly repairing injured tissue [106]. Fibroblast can be induced to acquire the myofibroblast phenotype during wound repair. Then, the myofibroblast, by virtue of its ability to express high levels of a-smooth muscle actin, cytokines and extracellular matrix, is expected to play key roles in the post-traumatic inflammatory skin response [104].

Several days after injury a subset of wound fibroblast can differentiate into myofibroblast which are responsible for repopulating the wounded area in parallel to angiogenesis, thus forming the granulation tissue [2,21,99]. Also, myofibroblast are responsible for wound contraction. However, to highlight the fact that contractile cytoskeleton is not a feature of normal tissue fibroblast, Boris Hinz introduced the term "proto-myofibroblast" for stress fiber-containing, but a-smooth muscle actin-negative fibroblast that would consist of the early granulation tissue and preorganize the provisional extracellular matrix [106].

Gastrulation is the first major shape change of the developing embryo and cell-directed mechanical forces have a critical role [114]. Contraction of the actin cytoskeleton, driven by nonmuscle myosins and regulated by the Rho family GTPases, is a recurring mechanism for controlling morphogenesis throughout development, from gastrulation to cardiogenesis [115-117]. Visualizing the location of nonmuscle myosin by immunostaining strongly suggests the role of myosin in regulating tension and compression during gastrulation [116]. The presumed mechanical effect is that myosin is causing the outer or apical surface to contract. Individual mesoderm cells constrict apically and leave the epithelium in a process known as ingression. Simultaneously, a loss of tension around the inner surface is produced. This causes the cell layer to buckle inwards leading to invagination [115,116].

Granulation tissue is then repopulated with fibroblasts to produce a more densely collagenous extracellular matrix which is more akin to the matrix found in interstitial stroma [118]. Recently, it has been demonstrated that subcutaneous granulation tissue induced by a large foreign body is a source of adult stem cells. This granulation tissue is derived from a local resting stem cell pool, particularly from dedifferentiate pericytes [119].

Tissue remodeling requires the removal of granulation tissue and maturation of collagen is oxygen dependent. Indeed increasing wound oxygenation results in increased collagen deposition and tensile strength [120]. However, hypoxia benefits the expansion, differentiation, adhesion, growth factor secretion and regenerative potential of mesenchymal stem cells derived from subcutaneous adipose tissue [121].

The synthesis and deposition of extracellular matrix largely occurs in response to mechanical signals mediated via cell receptors [115,116], cytokines and growth factors [118]. Particularly, the extracellular matrix can bind to and release certain growth factors, i.e. FGF-2, VEGF and PDGF, thereby exerting direct control over their activity [118].

The notochord is an embryonic structure that could be a candidate for comparison with this phase of tissue remodeling. This anatomical structure of the embryo dominates early morphogenesis as a source of molecular signals i.e. growth factors, and later as a mechanical structure [122].

Degradation and remodeling of the extracellular matrix by proteases, particularly matrix metalloproteases (MMPS) is a key feature of re-epithelialization and tissue remodeling [17]. Interestingly enough, the differentiation of an antifibrotic myofibroblast phenotype in response to tissue injury could inhibit collagen production as well as fibroblast proliferation. The fact that fibrosis may be due to a loss in antifibrotic properties rather than due to the activation of a fibrotic process suggests that, in normal tissues, active mechanisms to suppress fibrosis may be constitutively important in maintaining tissue homeostasis [104].

The phenotypic changes suffered by the keratinocytes during re-epithelialization suggest a partial epithelial-mesenchymal transition (Figure 7). Following the completion of wound-repair, keratinocytes revert to their mesenchymal-like phenotype to epithelial phenotype [18,123]. During the immediate keratinocyte response to the injury, one of the principal transducer signals can be electrical, by depolarization and hyperpolarization of the plasma membranes. Free ion movement occurs suddenly through membrane pores, which can either be opened or closed in response to a great variety of gating mechanisms, including voltage gating [124].

A cut in the skin produces a current that can be detected. This "injury" potential represented the short circuiting of a transepithelial potential that generated electrical field vectors as current flowed from areas of high resistivity, namely, with intact tight junctions, to the cut, where resistivity is low. Thus, the short circuiting of transepithelial potentials can be sensed over 1-2 mm from the wound and will persist until repair and re-epithelialization is complete [124,125].

One to two days after injury, the migration of keratinocytes from the epidermis at the wound edge and from injured appendages is produced [18,21]. Epithelial cell migration requires the disassembly of desmosomes and hemidesmosomes, which provide anchorage of the basal keratinocytes with neighboring epithelial cells and the underlying basement membrane respectively [126]. This disassembly and keratinocytes migration require cross-talk between growth factors, MMPs, integrins and structural proteins. In addition to lamellipodia extension, basal keratinocytes leapfrog over the basal cell near the wound [126].

The keratinocytes that are behind the leading edge in larger wounds proliferate and mature and finally restore the barrier function of the epithelium [18,21,49]. This could involve the proliferation of epidermal stem cells [18]. The proteins involved in re-epithelialization include various extracellular matrix proteins and their receptors, proteases and cytoskeletal proteins. Growth factors that are known to stimulate wound re-epithelialization include hepatocyte growth factor (HGF), FGF, TGF-a and heparin-binding epidermal growth factor (HB-EGF). The signaling pathways initiated by these growth factors activate the transcription factor signal transducer and activator of transcription 3 (STAT 3) and activator protein (AP)-1, which help to regulate wound re-epithelialization [18].

It has long been known that chronic wounds are at risk for neoplastic progression. Chronic inflammation is a major risk for various types of cancer [127]. The risk of squamous cell carcinoma is markedly increased, suggesting that keratinocytes are especially vulnerable to malignant transformation [17]. The main difference between the migration of wound keratinocytes and cancer cells is the complete epithelial-mesenchymal transition that is frequently seen in cancer cells [128]. The epithelial-mesenchymal transition that is associated with cancer progression is considered a type 3 epithelial-mesenchymal transition [123]. Carcinoma cells undergoing a type 3 epithelial-mesenchymal transition lose all cell-cell contacts, acquire a fibroblast-like morphology and express mesenchymal marker proteins. These processes resemble those that are activated during early embryogenesis (type 1) [123], and after skin injury (type 2) [123,128].

There is now broad evidence that tumor cells depend on metabolic alterations for their continued growth and survival, and that these changes make cancer cells peculiarly addicted to the rapacious uptake of glucose and glutamine [129,130]. This means that glucose and glutamine supply most of the carbon, nitrogen, free energy and reducing equivalents needed to support cell growth and division [124,131]. Proliferating cells during tumor progression, immune response or wound repair, have a similar metabolic regulation that allows for maximizing their rate of anabolic growth and proliferation [131]. This type of cell metabolism directed at growth and proliferation, is also efficiently used during mammal embryogenesis [129]. This is why it has been proposed that the alterations in metabolic control during wound repair and tumorigenesis may result from reverting to an embryonic program [131]. If so, inflammation, a common process to wound repair, tumorigenesis and embryogenesis could have a trophic purpose for the cells [2,10,24,40,132,133].

### When emulating gastrulation is not enough to heal wounds by regeneration

In the current review we have proposed that the inflammatory response employed by the adult for wound repair could resemble the early phases of embryogenic development. This hypothesis is based on the comparison of the successive phases of the inflammatory response, particularly in the mechanisms that regulate the earliest steps, in amniote gastrulation (Figures 3 and 4).

Gastrulation is a developmental phase that delineates the three embryogenic germ layers: Ectoderm, endoderm and mesoderm. Haeckel coined the term gastrulation derived from the Greek word "*gaste*", meaning stomach or gut, that transforms the rather unstructural early embryo into a gastrula with several specific characteristics: The three primary germ layers are formed; the basic body plan is established, including the construction of the rudimentary body axes; and the cells assume new positions, allowing them to interact with cells that were initially not close to them [134].

In essence, gastrulation could be represented as the creation of an interstitial space that is successively infiltrated by molecules and cells, in a similar fashion as the inflammatory interstitium of the traumatized tissue. Therefore, during gastrulation, the extraembryonary mesodermis internalizes and occupies the space located between the amnion and the yolk sac. The primitive streak is a subpopulation of the epiblast in the organizing center for amniote gastrulation [135]. Once the initial primitive streak is established, germ layer formation begins. At the primitive streak, the epiblast cells undergo the epithelial-mesenchymal transition to form the primary mesenchyma between the amnion and the yolk sac [136]. Afterwards, this is followed by mesenchymal-epithelial transitions to create secondary epithelium as part of somitogenesis and further commitment and diversification of cells forming mesoendodermal structures [136,137]. The concept that fibroblasts are simple residual embryonic mesenchymal cells explains the incorrect and often interchangeable substitution of the term "fibroblast" for "mesenchymal cell" [137].

The vast arrangement of the mesenchyme around and between the developing amniotic and yolk sac cavities suggests an important role of the mesenchyma in orchestrating embryo development. Mesenchymal stem cells are a versatile group of cells derived from mesodermal progenitors and can be found in several fetal and adult tissues [138]. The amnion is comprised of two layers, an epithelial monolayer and a stromal layer. From the human amnion it is possible to isolate two cell types, amniotic epithelial cells, derived from the embryonic ectoderm, and amniotic mesenchymal stromal cells, originating from the extraembryonic mesoderm [139,140]. Mesenchymal stem cells isolated specifically from the amniotic membrane could differentiate into neuronal-like cells which are identified to secrete dopamine [141]. Cells derived of amniotic fluid also have a neuronal, dopaminergic phenotype [142]. These results allow for considering the amnion as an embrionary functional axis with strong neural potential [141,142]. In addition, mesenchymal stem cells derived from the amnion are a transplantable cell population with therapeutic potential for multiple central nervous system disorders, especially stroke [140]. Experimental and clinical studies have demonstrated that amniotic membrane transplantation has important biological properties including anti-inflammatory, anti-microbial, anti-fibrosis and anti-scarring, as well as low immunogenicity. It also favors re-epithelialization [139,140]. Amnion-derived multi-potent progenitor cells secrete a unique combination of cytokines and growth factors, known as amnion-derived cellular cytokine solution, which establish a communication network between mesenchymal and epithelial cells during embryo development. That is why using the amnion to accelerate wound healing through its functions has been proposed, which regulates migration, proliferation and differentiation of fibroblast and keratinocytes [143].

The extraembryonic visceral yolk sac in mammals is composed of two layers and the visceral endoderm, which is active in endocytosis/digestion and has large lysosomes and the underlying mesoderm layer [144]. In the embryonic mesoderm layer "blood islands" develop, supporting hematopoiesis and angiogenesis [36]. Also a major function of the yolk sac is associated with the accumulation of carbohydrates, proteins and lipids for embryo nutrition (*vitellum*) [145]. Particularly, the yolk sac plays a vital role in providing lipids and lipid-soluble nutrients to embryos during the early phases of development [145,146]. The yolk sac uses high-density lipoproteins (HDL) and very low-density lipoproteins (VLDL) as carriers to incorporate cholesterol from the material circulation and to transfer it to the embryonic side [145]. Interstitial lipid accumulation of cholesterol, a precursor molecule of many hormones, like aldosterone, corticoides, androgens, strogens and progesterone, may favor fluid infiltration and cell migration, proliferation and differentiation during embryo development [147].

**Figure 9:**
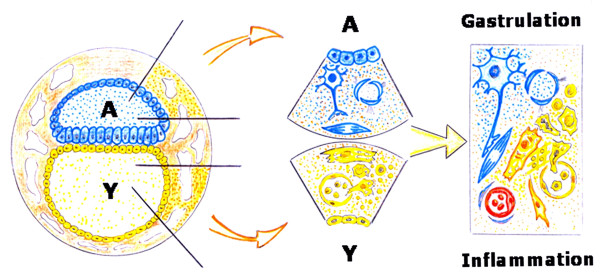
**The amniotic embryo**. The extraembryonic mesenchyma constitutes the walls of the amnion (A) and the yolk sac (Y). During gastrulation it could be considered that the extraembryonic mesenchyma internalizes with its functions: hydroelectrolitic control and neurogenesis in the amniotic side and angiogenesis and hematopoiesis in the yolk sac side. The internalization of the extraembryonic mesenchymal functions could integrate the amnion and the yolk sac original functions into the intraembryonic mesenchyma. Nonetheless, during the inflammatory response, the dedifferentiation process suffered by the tissues could re-express with clarity the primitive axes of the amnion and yolk sac.

The molecular and cellular contribution made by both embryo structures -the amnion and yolk sac- to the interstitial space located between them, that is the mesoderm, are essential for organogenesis. However, both in the amnion as in the yolk sac structures, the extraembryonic mesenchyma plays an important role (Figure 4). It could be assumed that both cavities, which are surrounded by epithelium, are controlled by an array of inductive and inhibitory signals originating from the adjacent extraembryonic mesenchyma. If so, the amnion wall, represented by the amniotic-mesenchymal-epithelial unit, plays the leading role in primitive interstitial hydroelectrolitic changes [28,31] and favors the development of a rudimentary neurogenesis [141,142]. In regards to the yolk sac wall, represented by the vitelline-mesenchymal-epithelial unit, it could exert functions fundamentally associated with hematopoiesis and angiogenesis [32,37]. (Figure 9).

**Figure 10:**
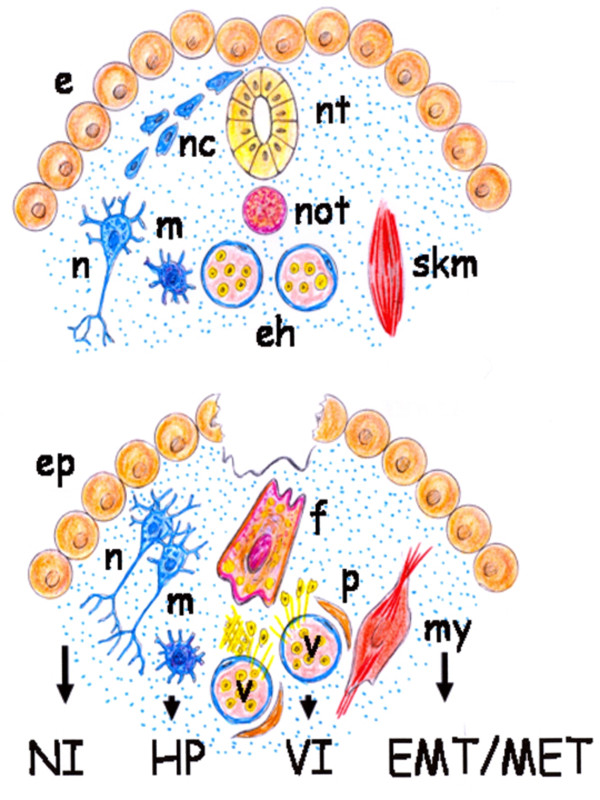
**Schematic representation of the gastrulation process (top) and of a skin wound (bottom)**. The hypothetical functional reactivation of embryonic development axes after a tissue injury would reproduce the embryonic biochemical pathways in the mature organism. e: epiblast; eh: endothelial-hematopoietic cell lineages; ep: epithelium; EMT/MET: epithelial mesenchymal transition/mesenchymal epithelial transition; f: fibroblast; HP: hyperpigmentation; m: melanocyte; my: myofibroblast; n:neuron; nc: neural crest cell; NI:neuronal inflammation; nt: neural tube; not: notochord; p: pericyte; skm: skeletal muscle; v:blood vessels; VI: vascular inflammation;

It could be accepted that these primitive functions are internalized during gastrulation to create the mesoderm. Thus, this germ layer would integrate the amnion- and yolk sac-related functions and would strengthen its functional ability compared to the extraembryonic mesenchyma (Figure 9). Therefore, the epithelial cells located near the dorsal midline of the neural tube undergo primary epithelial-mesenchymal transition and differentiate into many diverse derivates including neurons of the peripheral nervous system, glial cells and pigment cells, i.e. melanocytes [136,148]. In turn, the mesoderm and the endoderm contribute cells to other tissues of the developing animal. Thus, the yolk sac is an early source of hemangioblast, a common precursor of endothelial and hematopoietic cell lineages, and the cells of the visceral endoderm constitute a subpopulation of cells within the developing gut tube and therefore, have functions involved in digestion [149-151] (Figure 10).

In the adult organism, many pathways that play an essential role during embryo development are inactivated later in life although some of them may be transiently expressed during adult repair [152]. This hypothetical ability of the tissues to involute or dedifferentiate could be an effective defense mechanism against injury since it could make retracing a well-known route using the appropriate mechanisms for their repair. However, it is possible that the great dedifferentiation reached by the traumatized tissues has been underestimated. That is why in the current review we have considered that wound repair would require the upregulation of signaling pathways characteristic of the extraembryonic mesenchymal function, as well as of its posterior embryonary internalization during gastrulation. If so, emulation by the wounded tissue of the extraembryonic mesenchymal functions perhaps requires retracing the mechanisms that produce and distribute the extracellular fluid, including the amniotic fluid. These extraembryonic mesenchymal functions induce and regulate the neurogenic amniotic phenotype and activate the yolk sac-lipid metabolism associated with hemangioblast differentiation. It could be proposed that traces of these functions, based on stem cell lineages, are developed in the early phases of the post-traumatic inflammatory response (Figure 2) and would be expressed mainly in the interstitial space of the tissues (Figures 6 and 7).

As in the gastrulation process, it is possible that the primitive functions of the extraembryonic mesenchyma, whose re-expression is induced by the wound, would be internalized by the adult body. But, this mechanism would produce a serious loss of its health. So, if embryonic developmental pathways, representative of its primitive life, would re-emerge in the adult organism, they would take the place of post-natal specialized functions, impeding or hindering its actual life.

Nonetheless, according to the proposed hypothesis, the failure of adult skin regeneration after a wound suggests the need of researching those factors and mechanisms that must be upregulated and those that must be reduced or even avoided to efficiently reproduce the early embryonic conditions for development. Understanding the process that gives rise to early embryonic development may lead to advances in wound therapies. Perhaps, the mechanisms of scarless wound healing in the fetus are related to this supposed ability to maintain the memory of their origin while undergoing reprogramming events that could allow them to re-enter the embryonic programs of tissue formation to obtain the regeneration after injury [153,154].

### Mast cells as executors of the pathological axis of inflammation: guardians when not terrorist

Mast cells are ubiquitous in the interstitial spaces of the body and normally reside in the connective tissue close to blood vessel nerves and epithelia [155]. Current evidence suggests that mast cells exert their role, in all the inflammatory response phases, and that is why they are an ideal candidate for playing a role in wound healing [156] (Figure 10).

Particularly, mast cells could orchestrate the inflammatory response inducing the expression of embryonic programs of tissue formation. Mast cells are derivatives of hematopoietic progenitor cells that migrate into virtually all vascularized tissues, where they complete their maturation. Upon activation their "plasticity" allows them greater flexibility and diversity in term of responsiveness to meet the requirements of the inflammatory response in which these cells are involved [155]. In this sense, activated mast cells can induce the expression of the proposed neurovascular axes of the inflammation. Thus, mast cells secrete numerous biogenic monoamines, i.e. histamine, serotonin, and nociceptive molecules that can sensitize sensory neurons which further activate mast cells by releasing neurotransmitter or neuropeptides, i.e. acetylcholine, neurotensin, substance P and somatostatin [157,158]. Mast cells are also the source of many biologically active mediators involved in the process of neovascularization [155,159]. Mast cells are topographically associated with microvessels and their number rises in angiogenesis-dependent events such as inflammation [159,160]. The role of mast cells in angiogenesis is mediated by the release of their stored substances to a variety of stimuli [155,160,161]. Mast cell mediators include histamine, chymases, cytokine and growth factors like PDGF and VEGF, all of which exhibit pro-angiogenic properties [159-162]. In addition, post-traumatic inflammation is a strong pro-hematopoietic stimulus. Mast cells are one of the cells that produce pro-inflammatory pleiotropic mediators that induce hematopoiesis [163]. Hematopoietic stem cells, in turn, give rise to a hierarchically organized set of progenitors for erythroid, myeloid, lymphoid and megakaryocyte lineages [36]. Particularly, neutrophils, monocyte/macrophages and T cells from the bone marrow are always present in the interstitial connective tissue during inflammatory response progression [17,21]. Mast cells infiltrating injured tissues, through the release of granulocyte-macrophage colony stimulating factor (GM-CSF), can act on the bone marrow requesting the inflammatory cells needed for repair. A polarized hematopoietic axis from the bone marrow up to the mesenchymal interstitial space of the injured tissue would be established (Figure 10).

Mesenchymal post-traumatic dedifferentiation induced by activated mast cells could favor the expression of extraembryonic mesenchymal associated functions. In this way, embryonary mesenchymal functional axes are again expressed in the injured tissue, favoring in turn neurogenesis, hematopoiesis and angiogenesis. But mast cells also could simultaneously activate the mechanisms associated with gastrulation and this would mean the internalization of the above mentioned mesenchymal functional axes. In this developmental process, generation of the mesoderm from the ectoderm, that is the epiblast, by epithelial to mesenchymal transition [164], is associated with mesoderm cell constriction, leading to invagination [115,116]. Therefore, it is not surprising that during wound healing the excessive mesenchymal cell production and matrix deposition predominate, as well as the coexistence of wound contraction, which requires the presence of mast cells and contractile fibroblasts [156]. It is possible that in the inflammatory responses in which mast cells predominate, i.e. allergic and autoimmunes [165,166], this primitive embryonic mesenchymal response predominates. This hypothetical comparison could help to better understand the pathophysiological mechanisms and the histological characteristics that this type of chronic inflammatory response develops, particularly, the type of bidirectional interactions established between epithelial and mesenchymal phenotypes [165,167].

Mast cells could also participate in the worsening of the inflammatory response when a noxious factor is associated [168]. Mast cells are components of the innate immune system that acts as sentinels stationed around blood vessels, including swelling, redness and leukocyte recruitment [169]. Histamine, serotonin, proteases and VEGF stand out among the mediators released by mast cells, which cause vasodilation, edema and exudation due to increased vascular permeability [155]. The persistence of edema induced by mast cell mediators could maintain hypoxia in the wounded tissue and consequently inflammation [120,169]. However, chronic interstitial edema could also induce mesenchymal dedifferentiation. If so, mesenchymal fibroblasts embedded in the interstitial fluid could migrate and preserve their proliferation potential.

Under conditions of long-lasting inflammation, wound healing is associated with excessive interposition of fibrotic tissue. Prolonged inflammation in wounds contributes to the development of fibroproliferative scars, in other words, keloids and hypertrophic scar, both with erithematous and increased mast cell density [101,102]. Interactions between mast cells and fibroblasts are paramount in the genesis of fibrosis [168]. Fibrosis is characterized by excessive matrix deposition and reduced remodeling. Often fibrotic lesions are associated with increased densities of mast cells [170]. It has been shown that mast-cell secreted factors, specifically PDGF, could contribute to inflammation-associated fibrosis, inducing the expression of osteopontin by wound granulation tissue fibroblasts [171]. The fibroproliferative scars constitute solid cords along the wound axis which are related to wound remodeling. This excessive stiffness of the repair tissue resembles the notochord in the early embryo and, if so, it could be an ancestral trace of the mesenchyme also related to gastrulation. Particularly, mesoderm creation includes the development of a solid structure with great stiffness essential for correct morphogenesis, that is the notochord, and which is precisely what dominates the chordates.

Growth factors pivotal for repair in mammalians are PDGF/VEGF. In general, fibroblasts could respond in a pathological fashion to PDGF/VEGF promoting fibrotic tissue scarring [17]. Inflammatory cells, such as activated macrophages and mast cells, can produce inflammatory mediators that promote up-regulation of PDGF receptors on mesenchymal cells. As a result, PDGF-mediated proliferation of mesenchymal cells may be a hallmark of all chronic inflammation [172]. However, important developmental roles for PDGF and their receptor-like proteins have also been demonstrated in mammalian and non-mammalian vertebrates. Several observations suggest that PDGF has early developmental functions, particularly during gastrulation. PDGF receptors expression occurs in the developing mesoderm, mediating mesoderm cell migration, thus when the PDGF receptor is inhibited, the mesodermal cell detaches from the ectoderm and undergoes apoptosis [172,173]. PDGF signaling has an evolutionary conserved role during gastrulation and it has been implicated in neural and functional development as well as in the early differentiation of hematopoietic/endothelial precursor [172].

If we hypothesize that during wound healing a process similar to the embryonic gastrulation with internalization of the primitive extraembryonic functions is developed, we could deliberate about the original nature of these functions and their corresponding mediators. Therefore, it could turn to the mast cell functions and mediators because of its ubiquity during all the phases of the inflammatory response [156]. Mast cells are able to take up, store and release a variety of biogenic amines through which it is hypothesized that they participate in inflammatory reactions, mainly monoamines like histamine and serotonin [157]. These monoaminergic systems play a variety of roles in animals as neurotransmitters, autocrine and paracrine factors and hormones. However, also the venoms of many different animals, i.e. coelenterates, octopus, scorpions, centipedes, insects, amphibians and snakes contain histamine, in addition to other monoamines [174]. Also, the most ubiquitous effect of venoms on monoaminergic systems is venom-induced release of histamine from mast cells [174,175].

Likewise, the venoms of some species of coelenterates, octopus, spiders, scorpion, cone snails, centipedes, insects, amphibians and snakes contain serotonin [174]. It is thought that the most common function of both histamine and serotonin in venom is to produce pain and paralysis in the prey. Therefore, histamine and serotonin, characteristic mast cells mediators, are venom developed for both offensive and defensive purposes [174,175].

Examination of the effects of venoms on monoaminergic systems points out the great diversity of venom effects and also the cases of evolutionary convergence. For example, the venoms of scorpions, spiders and jellyfish cause a catecholamine storm in the victim [174,176-178].

A common feature of the *Phylum Cnidaria*, i.e. hydroids, anemones, corals and jellyfish, are tentacles with stinging cells, i.e. nematocytes or cnidocytes, which contain a nematocyst discharge mechanism. The nematocyst neurotoxins can paralyze and often kill the small prey, which are their food [178,179]. Both mechanical and chemical stimuli cause nematocysts to be discharged suggesting the involvement of both chemoreceptors and mechanoreceptors in the discharge process [179].

It is possible that during the evolution, these invertebrate armaments -defensive and offensive- were internalized into in the mesenchyma. If so, an evolutive advantage was associated with the risk of self-poisoning when the body suffered an injury that caused the cells to be stuffed with toxic products, i.e. the mast cells. Even psychological stress [180] because of the mutual association between mast cells and nerves [157] is a potential cause of mast cell inflammatory activation [155]. Interactions between the nervous system and the immune system, i.e. mast cells, are increasingly recognized as important in the pathophysiology of inflammation, including itch [181].

**Figure 11:**
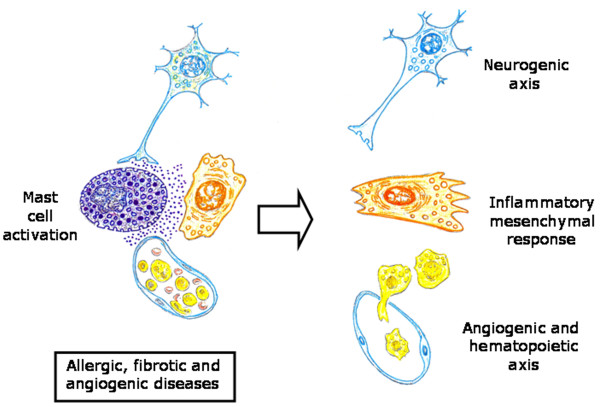
**Mast cell key role in orchestrating the inflammatory axis**. Upon activation, mast cells could induce the expression of both inflammatory axes, the neurovascular axis and the mesenchymal axis.

Mast cells exhibit phenotypical and functional heterogeneity in different anatomical sites [155]. Two types of mast cells have been identified in rodents, connective tissue-type mast cells (CTMC) and mucosal mast cells (MMC) [155,182]. Particularly, fetal skin-derived mast cells had many characteristic features of CTMC [182]. Mast cells are being preferentially localized at host-environment interface [155], although it is also accepted that they occupy strategic locations with respect to the basic structural division of the tissues and organs in the parenchyma, i.e. MMC and stroma, i.e. CTMC. This distribution suggests the involvement of the mast cell subtypes in the differentiation of the tissue structure. Perhaps thanks to these privileged locations in organs and tissues, mast cells may participate in the pathogenesis of a wide array of diseases, including allergic, inflammatory, angiogenic and fibrotic diseases [163,168,182]. In these anatomical locations mast cells, as interstitial cells, could exert a key role in modulating the epithelial-mesenchyma interaction, both in the physiological as in the pathological status (Figure 11).

## Conclusion

The ability of the tissues to involute or dedifferentiate could represent a return to early stages of development. Therefore, it could form an effective defense mechanism to escape death after injury since it could make retracing a well-known route possible, i.e. the initial phases of embryonic development during the evolution of the inflammatory response. Particularly, the up-regulation of signaling pathways during gastrulation, in which the internalization of extraembryonary functions would be made, could help explain the meaning of the diverse and complex mechanisms expressed by the mature organism in response to skin injury.

If so, even the embryonary mechanisms to generate pigment cells could be responsible of the post-inflammatory hyperpigmentation with higher concentrations of melanocytes [183,184]. Nowadays, the underlying mechanisms and the individual variability showed for developing post-inflammatory hyperpigmentation are not well understood [184,185].

This amazing property of the cells to conserve a memory of their origin undergoing reprogramming events could allow them, post-injury, to re-enter the embryonic programs of tissue formation and obtain regeneration [186]. Generally, while the monopotent and tissue-committed stem cells were described in the adult tissue, stem cells with a pluripotent/multipotent capacity were thought to be restricted to the early embryonic stages. However, recent evidence challenged this idea by confirming the presence of pluripotent/multipotent stem cells in adult tissues and organs [39]. Particularly, adult human mesenchymal cell populations, such as skin fibroblasts, contain distinctly multipotent stem cells. These stem cells have the ability to generate the multiple cell types of the three germ layers, like ectodermal (e.g. neural marker-positive cells), endodermal and mesodermal lineage cells [187].

Interestingly enough, quiescent and active adult stem cells coexist in several tissues that have the ability to renew and regenerate, like bone marrow, intestinal epithelium and hair follicles, all of them placed in the vicinity of a mesenchymal cell [188]. This stem cell-mesenchymal cell relation takes us back to the initial epiblast mesoderm relationship during gastrulation [36]. Thus, during vertebrate gastrulation, the cell behavior is strictly coordinated in time and space by various signaling pathways. In vertebrates, the non-canonical Wnt/planar cell polarity (Wnt/PCP) pathway is a key regulator of convergence and extension movements, also involved in the internalization of mesodermal cells and their migration [189]. The name Wnt is derived from a combination of two homologous genes; Wg (the Drosophila wingless gene) and Int (the murine homologue mouse mammary tumor virus integration site 1 gene) [190]. Wnt represents a large morphogenic family of secreted lipid-modified glycoproteins that during embryogenesis controls multiple developmental processes [189,190] and during adult life regulates tissue maintenance and remodeling [188,190]. At the cellular level, Wnt signals coordinate changes in cellular metabolism favoring either a "quiescent metabolism" or a "proliferating metabolism" [188,190]. This is why it is tempting to speculate that common alterations in metabolic programming may accompany embryonic and/or stem cell differentiation and these may also be involved in adult tissue development and/or remodeling [190].

In summary, the post-traumatic inflammatory response could be considered a reaction of the body in which the mesenchymal cell plays a leading role during which the early events of embryonic development and particularly gastrulation are recreated. The mesenchymal cell due to its strategic and privileged location in the interstitial space, is able to induce the successive phases of inflammation. Therefore, the mesenchymal cell uses the mast cell, to induce the expression of neurovascular inflammatory axes that have an extensive corporal projection and whose activity converges in the wounded skin.

The correlation that can be established between the embryonic and inflammatory post-traumatic events suggests that the results obtained from the research about both great fields of knowledge must be interchangeable to obtain the maximum advantage in the daily care of patients' wounds.

## Abbreviations

AP1: Activator protein 1; ATP: Adenosin-triphosphate; BDNF: Brain-derived neurotrophic factor; CGRP: Calcitonin gen-related peptide; CTMC: Connective tissue mast cell; FGF-2: Fibroblast growth factor-2; GM-CSF: Granulocyte-macrophage colony stimulating factor; GTP-ASE: Guanidine triphosphatase; HB-EGF: Heparin-binding epidermal growth factor; HDL: High-density lipoproteins; HGF: Hepatocyte growth factor; MMC: Mucosal mast cell; MMPS: matrix metalloproteases; NGF: Nerve growth factor; PDGF: Platelet-derived growth factor; RICE: Rest, Ice, Compression and Elevation; RNS: Reactive nitrogen species; ROS: Reactive oxygen species; STAT3: Transcription factor signal transducer and activator of transcription 3; TGF: Transforming growth factor; VEGF: Endothelial growth factor; VIP: Vasoactive intestinal peptide; VLDL: Very low-density lipoproteins; WNT/PCP: Wnt/planar cell polarity pathway.

## Competing interests

The authors declare that they have no competing interests.

## Authors' contributions

All the authors conceived, discussed, wrote and approved the manuscript.
